# Supplementary material for: Supplementation of Extender with Melatonin Improves the Motility, Mitochondrial Membrane Potential, and Fertilization Ability of Cryopreserved Brown-Marbled Grouper Sperm
Source: Animals (Basel). 2024 Mar 24;14(7):995. doi: 10.3390/ani14070995 (PMC11010917; doi:10.3390/ani14070995)
Supplement: Supplementary file 1 [file animals-14-00995-s001.zip › animals-2914375-supplementary.pdf]

1. Normality test

Table S1. The results of Shapiro-Wilk test of Motility parameters.

Melatonin	TM (%)		PM (%)		VCL (μm/s)		VSL (μm/s)		VAP (μm/s)	
(mg/mL)	W	*p*	W	*p*	W	*p*	W	*p*	W	*p*
0 (Control)	0.892	0.285	0.880	0.131	0.910	0.243	0.942	0.540	0.916	0.287
0.1	0.899	0.328	0.822	0.049	0.893	0.251	0.949	0.700	0.898	0.275
0.25	0.897	0.170	0.861	0.153	0.935	0.598	0.914	0.421	0.901	0.336
0.5	0.925	0.355	0.928	0.987	0.899	0.182	0.944	0.566	0.977	0.950

TM, total motility; PM, progressive motility; VCL: curvilinear velocity; VSL: straight linear velocity; VAP: average path velocity. The percentage data had been transformed by arcsine square root before the test. When $p>0.05$, this group of data is considered to be in line with normal distribution.

Table S2. The results of Shapiro-Wilk test of viability, cell apoptosis, MMP and hatching rate.

Melatonin	Viability (%)		Apoptosis rate (%)		High MMP (%)		Hatching rate (%)	
(mg/mL)	W	*p*	W	*p*	W	*p*	W	*p*
0 (Control)	0.996	0.877	0.891	0.358	0.958	0.606	0.963	0.630
0.1	0.973	0.693	0.963	0.629	0.996	0.875	0.884	0.336
0.25	0.957	0.603	0.919	0.449	0.951	0.572	0.892	0.359
0.5	0.975	0.700	0.994	0.850	0.862	0.272	0.806	0.129

The percentage data had been transformed by arcsine square root before the test. When $p>0.05$, this group of data is considered to be in line with normal distribution.

2. Correlation analysis

Table S3. The results of Pearson's correlation coefficient test between individual parameters.

		TM	PM	VCL	VSL	VAP	Viability	Apoptosis rate	High MMP	Hatching rate
TM	PCCs	1	0.29	0.846**	-0.399*	0.047	0.484	-0.107	0.351	0.326
	p	-	0.097	0.000	0.019	0.792	0.111	0.264	0.264	0.302
PM	PCCs	-	1	0.197	0.226	0.333*	0.513	0.363	0.323	0.323
	p	-	-	0.236	0.173	0.041	0.088	0.246	0.306	0.305
VCL	PCCs	-	-	1	-0.097	0.390*	0.429	-0.686*	0.499	0.743**
	p	-	-	-	0.545	0.012	0.164	0.014	0.099	0.006
VSL	PCCs	-	-	-	1	0.778**	0.347	-0.344	-0.071	0.149
	p	-	-	-	-	0.000	0.269	0.274	0.826	0.611
VAP	PCCs	-	-	-	-	1	0.480	-0.482	0.301	0.516
	p	-	-	-	-	-	0.114	0.113	0.342	0.086
Viability	PCCs	-	-	-	-	-	1	-0.382	0.278	0.253
	p	-	-	-	-	-	-	0.220	0.381	0.428
Apoptosis rate	PCCs	-	-	-	-	-	-	1	-0.326	-0.358
	p	-	-	-	-	-	-	-	0.301	0.254
High MMP	PCCs	-	-	-	-	-	-	-	1	0.638*
	p	-	-	-	-	-	-	-	-	0.025
Hatching rate	PCCs	-	-	-	-	-	-	-	-	1
	p	-	-	-	-	-	-	-	-	-

PCCs: Pearson correlation coefficient, *: P<0.05, **: P<0.01.
